# Molecular characterization of a glycerol-3-phosphate acyltransferase reveals key features essential for triacylglycerol production in *Phaeodactylum tricornutum*

**DOI:** 10.1186/s13068-016-0478-1

**Published:** 2016-03-12

**Authors:** Ying-Fang Niu, Xiang Wang, Dong-Xiong Hu, Srinivasan Balamurugan, Da-Wei Li, Wei-Dong Yang, Jie-Sheng Liu, Hong-Ye Li

**Affiliations:** Key Laboratory of Eutrophication and Red Tide Prevention of Guangdong Higher Education Institutes, College of Life Science, Jinan University, Guangzhou, 510632 China; Shenzhen Polytechnic, Shenzhen, 518000 China

**Keywords:** GPAT, Lipid, Diatoms, Biofuels

## Abstract

**Background:**

The marine diatom, *Phaeodactylum tricornutum*, has become a model for studying lipid metabolism and its triacylglycerol (TAG) synthesis pathway makes it an ideal target for metabolic engineering to improve lipid productivity. However, the genetic background and metabolic networks of fatty acid biosynthesis in diatoms are not well understood. Glycerol-3-phosphate acyltransferase (GPAT) is the critical enzyme that catalyzes the first step of TAG formation. So far, characterization of GPAT in marine microalgae has not been reported, especially at the level of comprehensive sequence-structure and functional analysis.

**Results:**

A GPAT was cloned from *P. tricornutum* and overexpressed in *P. tricornutum*. Volumes of oil bodies were produced and the neutral lipid content was increased by twofold determined by Nile red fluorescence staining. Fatty acid composition was analyzed by GC–MS, which showed significantly higher proportion of unsaturated fatty acids compared to wild type.

**Conclusion:**

These results suggested that the identified GPAT could upregulate TAG biosynthesis in *P. tricornutum*. Moreover, this study offers insight into the lipid metabolism of diatoms and supports the role of microalgal strains for biofuels production.

## Background

Renewable energy is one of the most effective solutions to address the carbon emission, energy security, and increased fuel consumption challenges that result in global warming and fossil fuel price concerns. These issues have prompted intensive interest in the capability of oleaginous microalgae to generate renewable oil sources which can be readily converted into biodiesel [1]. Microalgae can accumulate oil and are considered to be a promising feedstock for renewable biofuel production. For example, microalgae demonstrate much higher biomass productivity compared to higher plants and algal growth facilities could potentially be located in aquatic environments, which will not increase arable land concerns.

Although microalgae have reemerged as a potential 3rd generation feedstock for biofuel production, large-scale harvesting of microalgae is hampered by the lack of algal strains that can be selectively optimized for both high biomass generation capability and high TAG content [2]. One potential solution is to engineer robust oil-yielding microalgae by expressing the critical enzymes for TAG accumulation and to acquire a better overall understanding of lipid metabolic pathways in microalgae [3, 4].

In eukaryotes, TAGs are identified as neutral lipids that serve as the crucial storage form of energy. TAGs are the major feedstock for biodiesel production. TAG accumulation in microalgae is usually correlated to environmental stresses, such as high light intensity, high temperature, nitrogen limitation, and salinity [5, 6]. There are three major steps involved in TAG synthesis. Firstly, carboxylation of acetyl-CoA to form malonyl-CoA, which is the committing step of fatty acid biosynthesis in the plastid, secondly, acyl chain elongation in the plastid and cytosol, and finally, TAG formation in the endoplasmic reticulum (ER) [4]. The biosynthesis of fatty acids in chloroplast is catalyzed by two major, evolutionarily conserved enzymes, acetyl-CoA carboxylase (ACCase) and fatty acid synthase (FAS). The synthesized fatty acids are then esterified by glycerol-3-phosphate acyltransferase (GPAT) to glycerol 3-phosphate at the sn-1 position to form lysophosphatidic acid (LysoPA) [5, 7]. LysoPA is further catalyzed into phosphatidic acid (PA) by lysophosphatidic acid acyltransferase (LPAAT). The PA is then dephosphorylated by phosphatidic acid phosphatase (PAP) to form diacylglycerol (DAG). It has been reported that PA and DAG can also be formed in the chloroplast, where they serve as precursors for the synthesis of structural membrane lipids and neutral lipids [8, 9].

GPAT (EC 2.3.1.15) is considered as the initial enzyme for glycerolipid synthesis. In mammals, four GPAT isoforms have been identified [10–12]. In rats, hepatic glycerol-sn-3-phosphate acyltransferase 1 was overexpressed and caused hepatic insulin resistance, suggesting a role for lipid metabolites in the development of insulin resistance [7]. In humans, multiple isoforms of GPAT were expressed and differentially regulated in epidermis/keratinocytes [13]. In the model dicot plant, *Arabidopsis thaliana*, ten GPAT isoforms have been reported recently [14, 15]. These ten genes can be divided into three clusters. The first cluster is plastid-localized GPAT, which uses acyl-ACP substrates, and exhibits sn-1 acyl transfer regiospecificity [16]. The second cluster is GPAT9, which is located in the endoplasmic reticulum, and is enable to synthesize non-plastid glycerolipid [17]. The remaining eight GPATs do not play a role in the Kennedy pathway [14, 17]. Daubossy et al. reported that disruption of the UDP-glucose pyrophosphorylase gene in *Phaeodactylum tricornutum* resulted in increased TAG accumulation [18]. In our previous report, we successfully developed transgenic *P. tricornutum* with increased lipid accumulation by overexpressing type 2 diacylglycerol acyl transferase (DGAT) [19]. Similarly, overexpression of DGAT in *P. tricornutum* resulted in increased proportion of polyunsaturated fatty acids [20, 21]. However, no GPAT has been characterized in microalgae. Report of research employing metabolic engineering to increase lipid productivity in microalgae has been limited to date. In the present study, we first cloned a putative GPAT from the oleaginous marine diatom, *P. tricornutum*, and characterized its biological functions. This study will enable the use of genetically improved microalgal strains for industrial biofuel production.

## Results and discussion

### Analysis of GPAT sequences

**Fig. 1:**
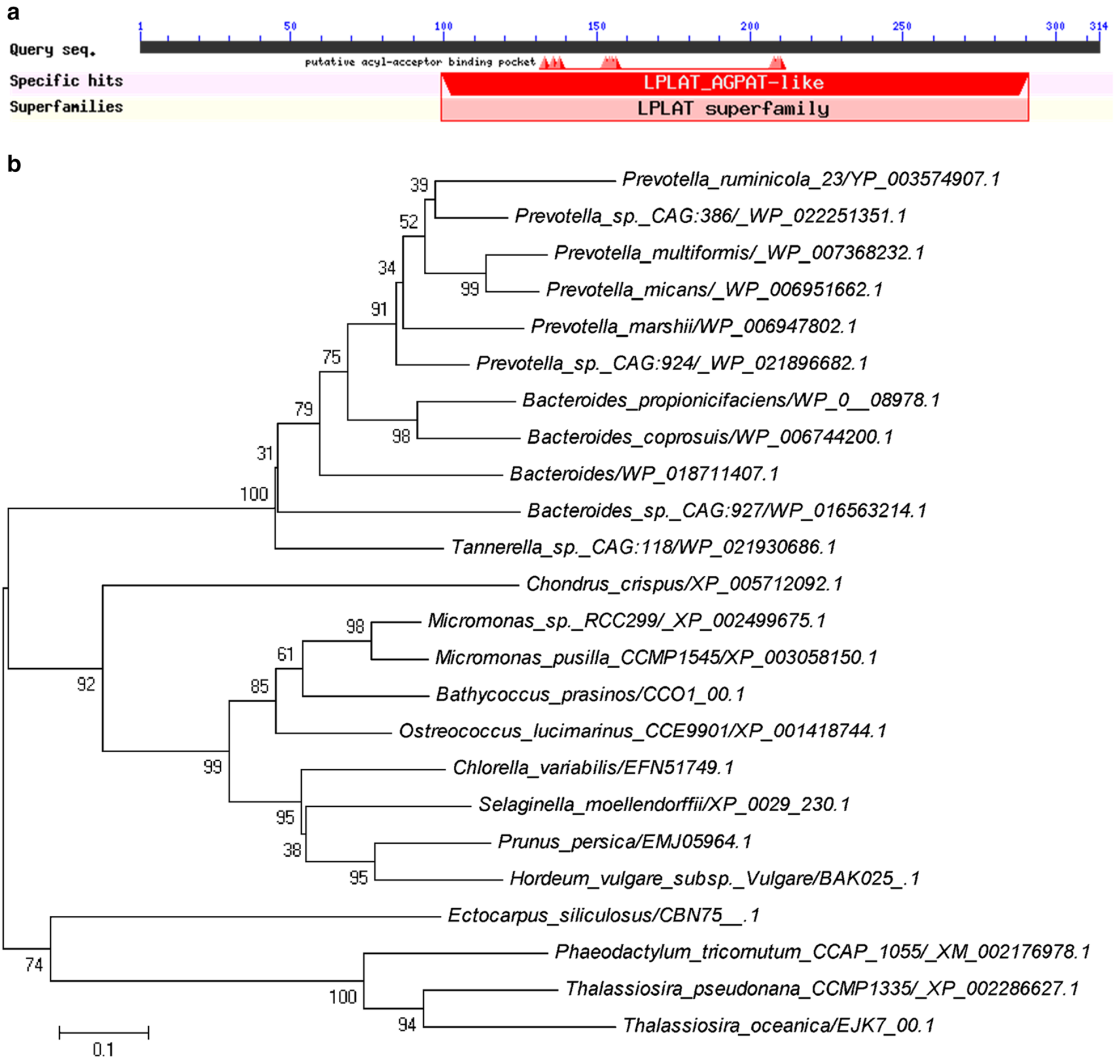
Conserved domain search and phylogenetic relationships of GPAT. **a** The conserved domain of LPLAT-AGPAT-like domain was detected. **b** Amino acid sequences of GPATs from various species were analyzed with software MEGA6

Conserved amino acid sequences for GPAT orthologs from various species were cataloged and used for BLAST interrogation of the *P. tricornutum* genome (National Center for Biotechnology Information—NCBI, http://www.ncbi.nlm.nih.gov/). Only one putative GPAT (PHATRDRAFT_42446, annotated as hypothetical protein) with high similarity to the consensus of GPAT was retrieved. Interestingly, PHATRDRAFT_42446 was annotated as a hypothetical protein and 1-acylglycerol-3-phosphate *O*-acyltransferase [EC:2.3.1.51] in KEGG (http://www.genome.jp/kegg/). The 315-amino acid sequence of this PtGPAT was predicted to contain a LPLAT_AGPAT-like domain spanning amino acid 100–292 (Fig. 1a), with a putative acyl-acceptor binding pocket predicted by a BLAST conserved domains search (http://www.ncbi.nlm.nih.gov/Structure/cdd/wrpsb.cgi). The phylogenetic relationships of GPAT with orthologs from various species were analyzed with the MEGA5 software using the neighbor-joining method. As shown in Fig. 1b, GPAT orthologs from 24 species were clustered into three groups. GPAT from *P. tricornutum* was closely aligned with orthologs from the marine diatoms, *Thalassiosira pseudonana* and *Thalassiosira oceanica*. Moreover, they were clustered into the same group with the marine brown alga, *Ectocarpus siliculosus*. This group was separated from the other two groups consisting of micro- and macroalgal origins. These results suggest that PtGPAT was well conserved during evolutionary development, having close relationships with other diatoms and brown algae, while remaining far from other species.

### Construction of transformation vector and generation of transgenic *P. tricornutum*

**Fig. 2:**
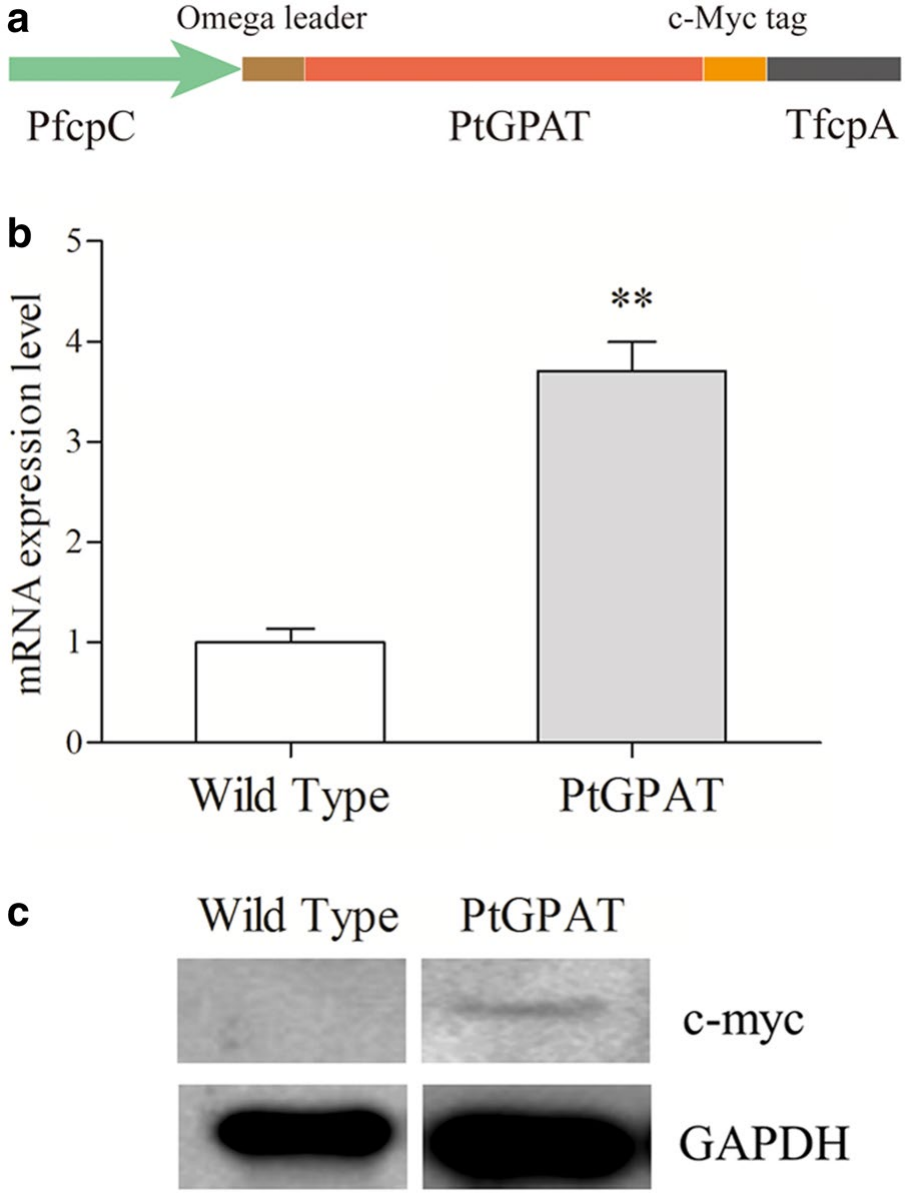
Expression of GPAT in *P. tricornutum.* **a** Schematic map of the PtGPAT expression cassette in pHY-PtGPAT. **b** Expression of GPAT transcripts measured by qPCR; β-actin was used as internal reference gene. Significant difference between control and treatment groups is indicated at *P* < 0.05 (*) or *P* < 0.01 (**) level. Each value represents mean ± SD (*n* = 3). **c** GPAT protein expression detected by Western blotting with an anti-c-Myc antibody; GAPDH was used as internal reference protein

*PtGPAT* cDNA was fused between the fucoxanthin chlorophyll a/c binding protein (fcp) fcpC promoter and fcpA terminator of *P. tricornutum*. Furthermore, an Omega leader sequence was added before the coding region of *PtGPAT* for enhancing its translation (Fig. 2a). A c-Myc tag was also fused to the *C*-terminal of PtGPAT. Hence, the expression of heterologous PtGPAT could be followed by Western blot analysis using anti-Myc antibody. *CAT* (chloramphenicol acetyltransferase) was used as selection marker gene. After electroporation, transformed algal cells were selected and grown for at least four successive subculture cycles under chloramphenicol (250 µg mL^−1^) selection followed by molecular characterization. The integration of the PtGPAT expression cassettes in the transformed *P. tricornutum* cells was confirmed by single-cell PCR using *CAT* primers. An expected PCR product of 0.7-kb PCR was detected in the transgenic microalgae, whereas no band was present in the wild-type strain (data not shown). These results suggested that the expression vector was successfully introduced into *P. tricornutum*. Several independent transgenic cell lines have been initially screened in terms of growth and neutral lipid content. To consider the performance of integrated characteristics, only the most remarkable transgenic line was discussed in the following sessions.

The relative transcript level of *GPAT* in transgenic lines was determined by qPCR using β-actin as a reference gene. Transcript abundance of GPAT was significantly increased by 3.7-fold in the selected transgenic line compared to wild type (Fig. 2b). To confirm the expression of PtGPAT protein, Western blot analysis was performed with an anti-c-Myc antibody. A specific cross-reacting band in accordance with the expected molecular weight was detected in the transgenic cells, whereas no analogous band was observed in the wild-type strains (Fig. 2c), indicating that the introduced PtGPAT was transcribed and also translated in the transgenic cells.

### Growth characteristics and photosynthetic performance of transgenic microalgae

**Fig. 3:**
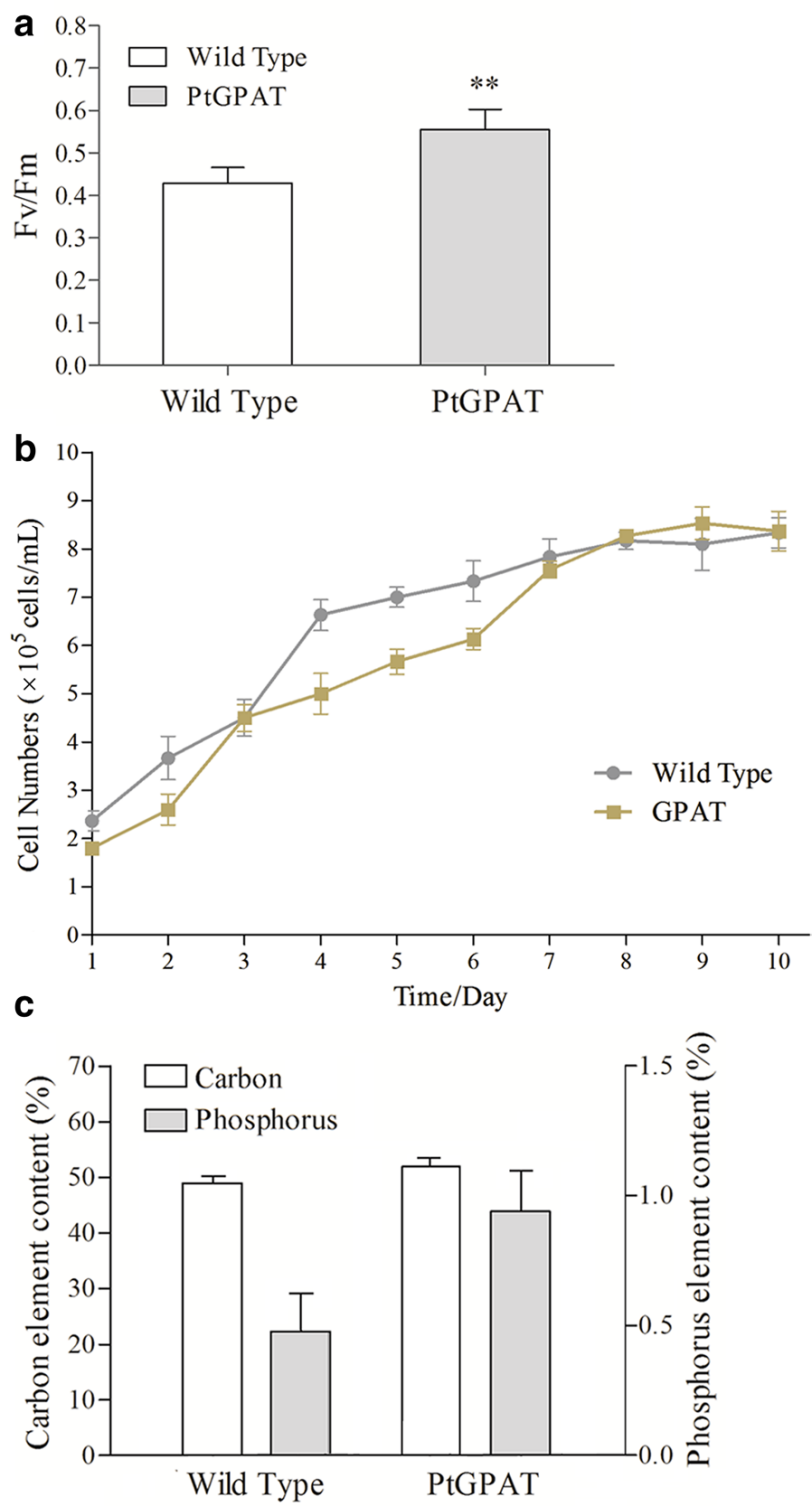
Photosynthesis performance and growth curve of *P. tricornutum.* **a** Fv/Fm showing photosynthesis activity; **b** growth curves of transgenic and wild-type microalgae. Significant difference between control and treatment groups is indicated at *P* < 0.05 (*) or *P* < 0.01 (**) level. Each value represents mean ± SD (*n* = 3). **c** Analysis of elemental content including carbon and phosphorus

Photosynthesis activity was measured to indicate the photosynthetic performance and acclimation status of transgenic *P. tricornutum.* The chlorophyll fluorescence parameter, Fv/Fm, was monitored for algal cultures in the stationary phase (Fig. 3a). Fv/Fm is the maximum photochemical quantum yield of PSII reaction center to reflect the light energy conversion efficiency of photosynthesis [22, 23]. A significant increase in Fv/Fm was detected in transgenic microalgae compared to wild type. It has been reported that the unsaturation of membrane fatty acids by the action of GPAT could result in the protection of the photosynthetic mechanism from photo-inhibition in tobacco and cyanobacteria [24].

In order to correlate the growth characteristics with the GPAT overexpression, we analyzed the growth curve for both transgenic and wild-type cells. As shown in Fig. 3b, transgenic and wild-type cells showed only a slight variation in growth curve and essentially reached stationary phase in a similar way. The results suggested that the GPAT overexpression did not have an apparent effect on the growth and biomass accumulation of transgenic microalgae.

Elemental analysis of carbon and phosphorus in *P. tricornutum* were further performed. As shown in Fig. 3c, transgenic cells showed a slightly higher carbon content than wild type. This increase of carbon content correlated well with the elevated carbon assimilation reflected by the higher photosynthetic performance. Phosphorus is the major constituent of the phospholipids which comprise cellular membranes. Up to a twofold increase in phosphorus was detected in transgenic microalgae. This increase in phosphorus content could reside in the phospholipids which form the membranes of oil bodies increased in transgenic cells. In some microalgal species, phospholipids in plastidial membranes could be recycled through membrane remodeling and used as source material for storage neutral lipid synthesis during certain stress conditions, such as nitrogen limitation [25, 26].

### Lipid productivity analysis

**Fig. 4:**
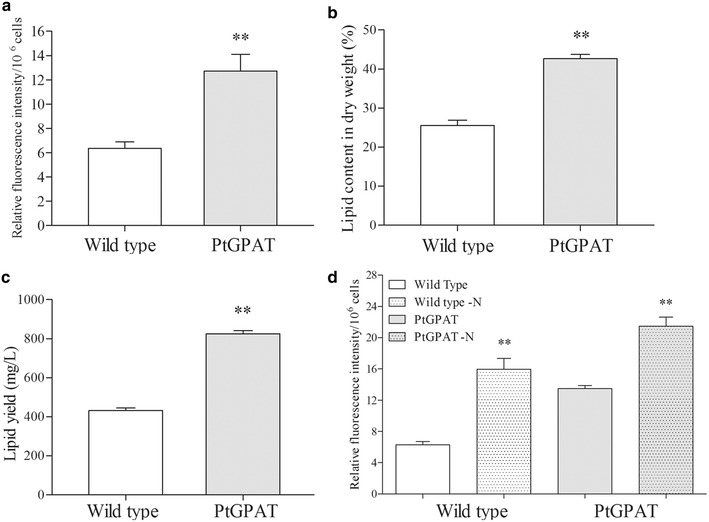
Lipid productivity in *P. tricornutum.* **a** Neutral lipid content as per 10^6^ cells determined by Nile red staining. **b** Total lipid content in dry cell weight determined by gravimetry. **c** Total lipid content as per culture volume. **d** Neutral lipid content under N-deprivation. Significant difference between control and treatment groups is indicated at *P* < 0.05 (*) or *P* < 0.01 (**) level. Each value represents mean ± SD (*n* = 3)

Neutral lipid content was first determined by Nile red staining in the stationary phase. Transgenic microalgae increased twofold compared to wild type (per 10^6^ cells) in the stationary phase (Fig. 4a). The neutral lipid content in dry cell weight (DCW) was further determined by conventional gravimetric measurements. The DCW per 10^9^ transgenic cells was measured to be 30.53 mg and the lipid yield was 12.21 mg. Correspondingly, the neutral lipid content was 42.6 % (DCW) in the transgenic microalgae, whereas it was 25.5 % in wild type (Fig. 4b). In terms of industrial production, the lipid productivity per culture volume is an important factor. As shown in Fig. 4c, wild-type cells were determined to contain a total lipid content of 480 mg L^−1^ culture, whereas transgenic cells reached 810 mg L^−1^ culture in the stationary phase. These results indicated that GPAT overexpression markedly improved the lipid productivity in diatom cells.

Since nitrogen deprivation can stimulate lipid production in some microalgae species, diatom cells in the stationary phase (7 days after subculture) were also subjected to nitrogen-deprivation treatments for 48 h. As shown in Fig. 4d, upon nitrogen-deprivation treatment, the neutral lipid content increased by 2.45-fold in the wild type and by 1.59-fold in the transgenic cells. Thus, the final neutral lipid content in transgenic cells was 1.34-fold that of the wild type.

**Fig. 5:**
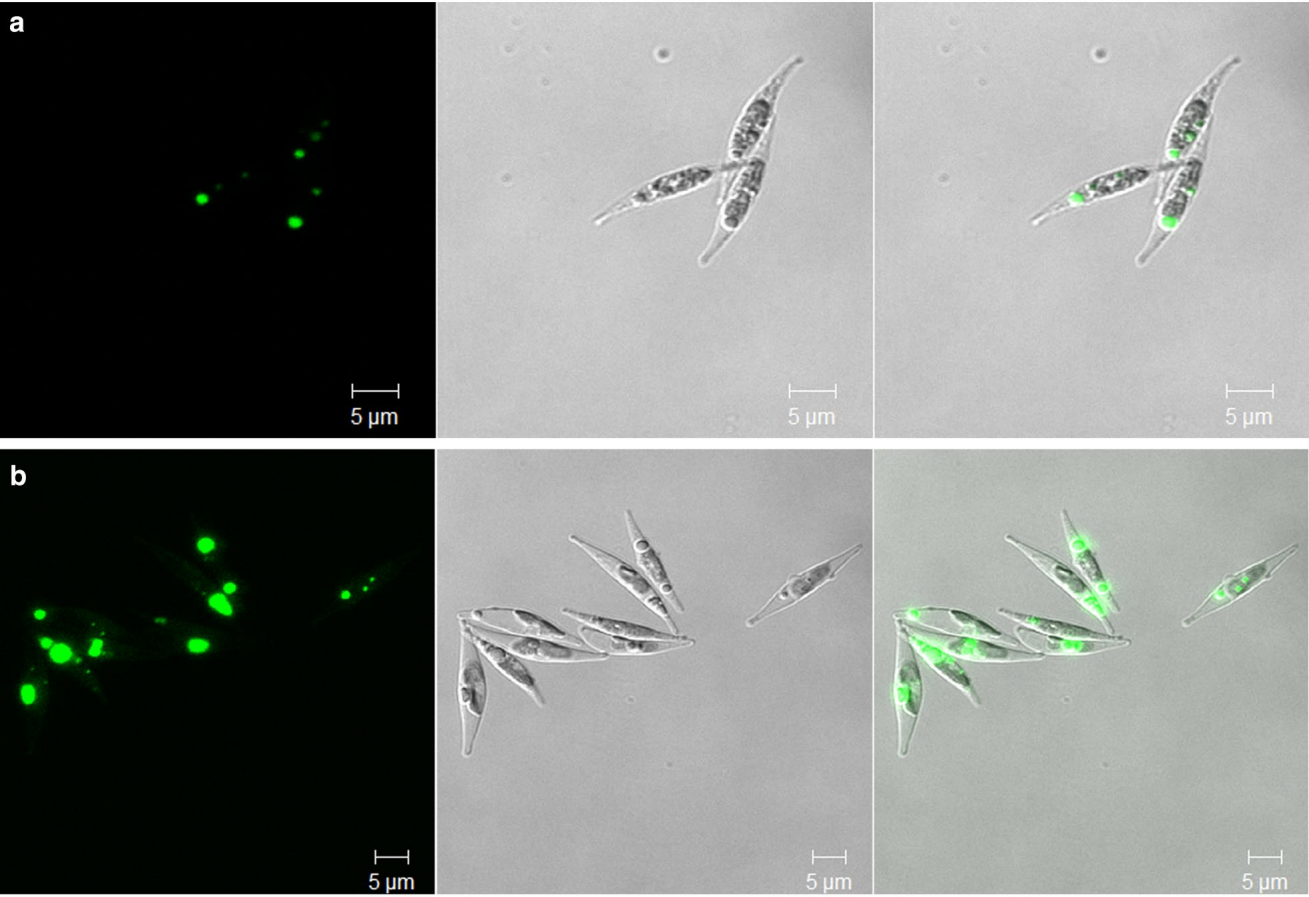
Morphological observations of the cell and oil bodies. Microalgal cells were stained with Nile red and observed under a laser-scanning confocal microscope. **a** wild type; **b** transgenic cells. *Left panel* fluorescence of oil bodies; *middle panel* differential interference contrast (DIC); *right panel* overlay image. *Bar* = 5 µm

To further examine the lipid accumulation in diatom cells, oil bodies were observed by Nile red staining under a confocal microscope. As shown in Fig. 5, the numbers of oil bodies were similar in both transgenic and wild-type cells, whereas the volume of oil bodies in transgenic cells was considerably increased and larger than in wild type. The larger volume of oil bodies that appeared in *P. tricornutum* overexpressing GPAT was consistent with the increase in neutral lipid content observed.

### Changes in fatty acid composition regulated by overexpression of GPAT

In order to detect the effect of GPAT overexpression on fatty acid composition in transgenic and wild-type microalgae, cells in the stationary phase were analyzed by GC–MS analysis. Interestingly, significant differences in fatty acid composition were detected between transgenic and wild-type cells (Table 1). Proportions of saturated fatty acids (SFA) and monounsaturated fatty acids (MUFA) decreased by 35 and 12 %, respectively; but polyunsaturated fatty acids (PUFA) increased by 41 %. Notably, C16:3 fatty acid was 52 % higher than that of wild-type cells, and C20:5 was almost 40 % higher; however, C16:0 was about 45 % lower. The changes in fatty acid composition were largely due to dramatic increases in PUFAs, which are generally the abundant fatty acids in *P. tricornutum* [27]. These increases correlate with losses of SFAs and MUFAs. Thus, the altered content of unsaturated fatty acids in transgenic diatoms was due to the activity of overexpressed GPAT. Yokoi et al. reported that expression of *Arabidopsis* GPAT resulted in increased content of unsaturated fatty acids in transgenic rice compared to wild type [28]. Expression of *Brassica napus* GPAT resulted in increased oil content in transgenic tobacco [29]. Iskandarov et al. reported that fatty acid content was increased in the transgenic green microalga, *Chlamydomonas reinhardtii*, expressing *Lobosphaeraincisa* GPAT and resulted in increased TAG accumulation [30]. Similarly, induced expression of *Helianthus annuus* GPAT in transgenic *Escherichia coli* resulted in increased unsaturated fatty acid content [31]. Accordingly, the proportion of unsaturated fatty acids showed an increase in the present study. For the industrial scale biofuel production process, an optimized biological source is a prerequisite. The transgenic marine diatom overexpressing GPAT reported here could be a promising biofuel source with high lipid content and unsaturated fatty acids.

**Table 1:** Changes in fatty acid composition in transgenic and wild-type microalgae

Fatty acid	Wild type (DW %)	GPAT (DW %)
C14:0	3.54	2.48
C15:0	0.20	0.11
C16:0	23.60	16.17
C18:0	7.35	5.93
C22:0	0.17	0.36
C24:0	0.29	0.85
SUM SFA	35.15	25.9
C16:1	25.08	26.85
C18:1	5.95	0.84
SUM MUFA	31.03	27.69
C16:3	4.19	6.41
C18:2	1.09	1.66
C18:3	0.08	0.04
C20:5	18.23	25.53
C22:6	0.81	0.87
SUM PUFA	24.4	34.51

### Subcellular localization of GPAT in *P. tricornutum*

PtGPAT was predicted to consist of two transmembrane helices by membrane topology prediction server HMMTOP (http://www.enzim.hu/hmmtop/server/hmmtop.cgi), spanning amino acid 9–23 and 56–80. Subcellular localization analysis was performed using prediction programs. A signal peptide was predicted in PtGPAT using TargetP 1.1 Server (http://www.cbs.dtu.dk/services/TargetP-1.1/) with a 0.754 possibility. WoLF PSORT Prediction (http://www.genscript.com/psort/wolf_psort.html) was used to further predict the subcellular localization, which suggested that the most likely location was the chloroplast. LocTree3 (https://rostlab.org/services/loctree3) also predicted the chloroplast location.

**Fig. 6:**
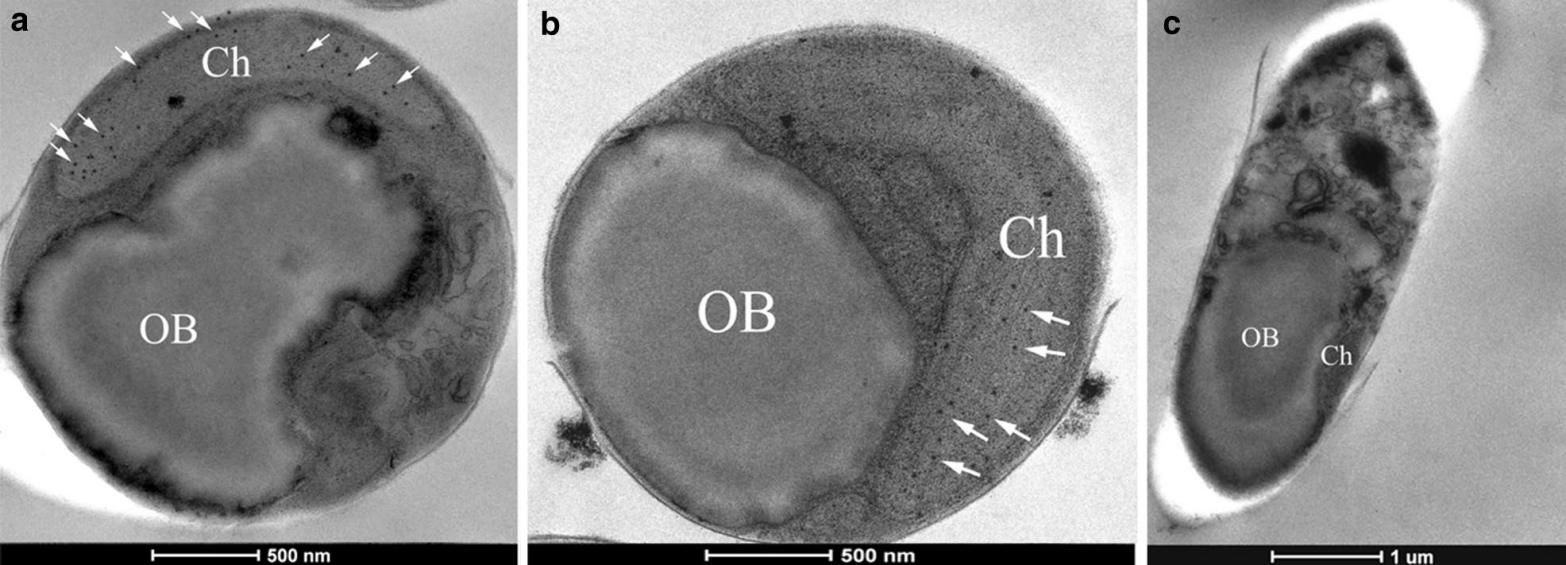
Subcellular localization of GPAT in *P. tricornutum.* The Myc-tagged PtGPAT was detected by immuno-gold labeling with Myc-specific antibody in the microalgal cell. Transverse sections of transgenic cell (**a** and **b**) and wild type (**c**) were photographed under EM. The *arrows* indicate the gold particles; *OB* oil body; *Ch* chloroplast; *Bars* = 500 nm in (**a** and **b)** and 1 μm in (**c**)

The subcellular localization of PtGPAT in diatom cells was further determined by immuno-electron microscopy. Gold particles were found predominantly in chloroplasts in PtGPAT-overexpressing transgenic cells (Fig. 6a, b), whereas no gold particles could be found in the wild type (Fig. 6c). These results indicated that PtGPAT was predominantly localized in the chloroplast, which is overall consistent to the software prediction.

Subcellular localization is a crucial attribute of a protein and indispensable to understanding the biological processes at the cellular level. Hence, predicted subcellular localization of proteins is fundamental to Systems Biology [32]. So far, the subcellular localizations of PtGPAT were determined to be varied in different organisms, including the cytosol, chloroplasts, or mitochondria. In 1988, cDNA of GPAT was first cloned in chloroplasts of squash cotyledon [33]. Some algal and higher plant GPAT proteins were also predicted in chloroplast using subcellular targeting localization prediction [5]. In sunflowers, abundant sn-glycerol-3-phosphate acyltransferase was identified in the chloroplast [31]. Subcellular localization prediction analysis in the arachidonic acid-rich microalga, *Myrmecia incisa* Reisigl H4301, demonstrated that GPAT might target the chloroplast [34]. Two glycerol-3-phosphate acyltransferase-like (GPAT-like) proteins from the tung tree, GPAT8 and GPAT9, were found to co-localize in the same ER subdomains [35]. Functional differences between ER-GPAT and mitochondrial (Mt)-GPAT in *Caenorhabditis elegans* demonstrated that Mt-GPAT was required for mitochondrial fusion [36].

In this study, GPAT was predicted to localize in the chloroplast and this notion was evidenced by immuno-EM. In photosynthetic organisms, fatty acid biosynthesis can take place in the chloroplast. GPAT, along with the acyl-(acyl carrier protein), catalyzes the first reaction of glycerolipid assembly in the chloroplast; the substrates for these enzymes are synthesized within the chloroplast [31]. By a series of sequential enzymatic reactions, once the fatty-acyl chain attains its chain length (C 16:0 or C 18:0), it can be esterified by GPAT to form LysoPA. LysoPA is added to subsequent acyl chains from LPAAT to give PA, which is dephosphorylated to yield DAG, the precursor for algal glycerolipids [5, 37, 38]. The free fatty acids from the plastid are transported to the chloroplast and ER, where they are attached to -CoA by long-chain acyl-CoA synthase. The same sequential reactions can occur in the microalgal chloroplast [37, 38].

## Conclusions

Considering that functional biochemical studies of key enzymes in microalgae have been lacking, development of recombinant microalgae based on metabolic engineering has been limited compared to work in higher plants. Although foreign gene expression in a few microalgae species has been achieved, the relatively lower expression level makes it imperative to develop effective industrial scale microalgae bioreactors. GPAT is the key enzyme in triglyceride biosynthetic pathway. This study focused on functional and molecular characterization of GPAT, as well as its role in enhancing the lipid accumulation in oleaginous microalgae. Overexpression of GPAT in transgenic *P. tricornutum* promoted the formation of oil bodies and stimulated twofold neutral lipid content with an overall increased productivity. These results highlighted the concept that a metabolic engineering approach could be the most effective method for achieving maximal enzymatic activity and thus maximal lipid productivity in microalgae. Using a genetic-metabolomic approach, it should be possible to successfully boost TAG storage levels in microalgae, thus increasing effective routes for biofuel production.

## Methods

### Algal culture conditions

The microalga strain, *P. tricornutum* (CCMP2561), was obtained from the National Center for Marine Algae and Microbiota. Microalgae were grown as batch cultures in flasks containing f/2 medium, which was filter-sterilized through 0.22 µm filters. Cultures in liquid medium or on the plate were grown at 21 ± 1 °C in an artificial climate incubator (Ningbo, China). Cool-white fluorescent tubes provided an irradiance of 200 µmol photons m^−2^s^−1^ under long-day light conditions (12/12 h light/dark regime). Cell numbers of the cultures were counted using Brightline Hemocytometer under microscope at the same time every day, in triplicates. Cell density was calculated according to Yang et al. [26].

### Cloning of putative GPAT and construction of expression vector

Genomic DNA of *P. tricornutum* was extracted using Universal Genomic DNA Extraction Kit Ver.3.0 (Takara, China). The full-length coding region of putative PtGPAT was cloned by PCR with primers designed according to genome sequence of *P. tricornutum* on JGI website. Primer pairs used were Pt49 (5′-ACCATGGACTTATCTACGGTTCAATCAG-3′) and Pt73 (5′-GTCTTATTCTTGTCGGAGTCGTTG-3′). The PCR product was cloned into pHY29 derived from Yao et al. [39] by overlap extension PCR cloning method [40]. In the resultant plasmid, a c-Myc tag (amino acid sequence: EQKLISEEDL) was fused at the C-terminal PtGPAT for the detection of its protein expression.

### Generation of transgenic *P. tricornutum*

*Phaeodactylum tricornutum* was transformed by electroporation with pHY29-GPAT following the previously developed protocol by Niu et al. [41]. The transformed *P. tricornutum* plates were incubated for 3 weeks under standard growth conditions and the emerging putative transformed colonies on the selection medium were counted. Survived colonies were picked and inoculated in liquid f/2 medium containing chloramphenicol (250 mg L^−1^), then subcultured for five more cycles on selection medium to obtain stable transgenic microalgae. Selected cells were subcultured in f/2 medium once a week supplemented with chloramphenicol (250 mg L^−1^). Cells in stationary phase were collected for molecular characterization.

To detect the integration of CAT, transformed microalgae were screened by single-cell PCR analysis with primers CAT-f and CAT-r [42]. PCR-confirmed cultures were then subjected to successive culture.

### Characterization of photosynthesis

The chlorophyll fluorescence parameter Fv/Fm (ratio of variable/maximum fluorescence), an empirically verifiable index of photosynthetic performance and acclimation status [22, 23], was used to characterize microalgal photosynthesis. Algal cultures were kept in the dark for at least 40 min. The chlorophyll fluorescence in the *P. tricornutum* culture was measured with a TD-700 fluorometer (Turner Design,USA) following the protocol by Macedo et al. [43].

### Elemental analysis

An aliquot of 200 mL transgenic and wild-type cultures (5 × 10^7^ cells mL^−1^) were harvested by centrifugation at 4 °C. The pellet was resuspended in 1 mL water and centrifuged at 4 °C. The pellet was resuspended in 1 mL water and centrifuged again. The pellet was baked at 60 °C for 48 h and was ground into powder. The powders were analyzed by using the instrument FE-SEM (Hitachi S-4800, Japan).

### Lipid analysis

Lipid content in transgenic *P. tricornutum*, transgenic microalgae with nitrogen deprivation, and wild type was measured by Nile red (Sigma, USA) staining according to Yang et al. [26]. Seven-day-old microalgae culture was collected and subcultured in −N and +N medium. For Nile red staining, 30 μL of solution of Nile red (0.1 mg mL^−1^ in acetone) was added into 3 mL of cell culture. Non-stained cultures were used as an auto-fluorescence control. Excitation and emission wavelength for detection were set at 530 and 580 nm, respectively. The fluorescence intensities reflect the relative neutral lipid content of stained cells.

The neutral lipid content of microalgae was also measured by gravimetric determination. An aliquot of 200 mL (6.55 × 10^7^ cells mL^−1^) of 7-day-old culture was harvested and lyophilized and the dry weight of microalgae was measured. To the lyophilized cells, 2 mL of methanol, 2 mL of chloroform, and 1 mL of 5 % NaCl were added and vortexed for 2 min. The mixture was centrifuged at 8000×*g* for 4 min, and the chloroform layer was collected. The same extraction procedure was repeated three times and the combined extracts were dried by N_2_ flow. The lipid residue was dried in oven at 60 °C and weighed to yield dry weight.

Lipid composition was analyzed by gas chromatography-mass spectrometry (GC–MS) according to Yang et al. [26]. Fatty acids were identified according to the equipped NBS spectrum library. The integrated peak areas were determined and calculated by normalization to obtain the percent contents of fatty acid composition.

### Analysis of GPAT expression by qPCR and Western blotting

The expression of the integrated GPAT from the stationary phase culture was analyzed by reverse transcriptase PCR, real-time PCR, and Western blot analysis. For Reverse Transcriptase PCR analysis, RNA was extracted from PCR-confirmed culture and wild-type culture using Takara RNA kit (Takara, Japan). RT-PCR was carried out with HiScript^®^ II Q RT SuperMix for qPCR + gDNA wiper (Vazyme, China) according to manufacturer’s instruction.

The expression of GPAT was quantified by quantitative real-time PCR (qPCR) using SYBR Green I PCR Master Mix (Bio-Rad, USA) on ABI PRISM^®^ 7500 Sequence Detection System (ABI, USA). PtGPAT was amplified using primers:

q49 (5′-ACGACAAGGTCGGAACAAAC-3′) and q73 (5′-TAAAGGCACCGTCCTTGAAC-3′). β-actin was used as reference gene. The threshold cycle (Ct) for each well was measured and mRNA levels of the PtGPAT were quantified after normalization to β-actin.

The expression of PtGPAT protein was examined by Western blot analysis with an anti-Myc antibody against the Myc-tagged PtGPAT. An aliquot of 200 mL microalgal culture (5 × 10^7^ cells mL^−1^) was harvested and total protein was extracted with Protein Extraction Kit as per the supplier’s instruction (KeyGEN, China). Protein concentration was estimated by Bradford protein assay (Bio-Rad, USA). Proteins were separated by electrophoresis by 10 % SDS-PAGE and electrotransferred to polyvinylidene fluoride membrane. The membrane was blocked with skimmed milk for 2 h at 4 °C. Thereafter, membrane was treated with anti-Myc antibody (1:3000; Sigma-Aldrich, USA). The anti-GAPDH antibody was used as reference (1:3000; Sigma-Aldrich, USA). HRP-conjugated goat anti-rabbit antibody (CST, USA) at a dilution of 1:5000 was used as secondary antibody. Membrane development was performed using chemiluminescent system.

### Morphological observation of *P. tricornutum* and subcellular localization of GPAT

To observe the cell morphology and oil bodies in *P. tricornutum*, cells were stained with Nile red fluorescence dye and observed under a fluorescence microscope. One-milliliter microalgae cells were stained with 10 μL of Nile red (0.1 mg mL^−1^ in acetone) and incubated in darkness for 20 min. A laser-scanning confocal microscope Zeiss LSM 510meta (Zeiss, Germany) with excitation wavelength of 488 nm and emission wavelength of 505–550 nm was used to observe the morphology of the stained cells.

Subcellular localization of PtGPAT was analyzed using Target P, WoLF PSORT, and LocTree3. In order to localize the expressed PtGPAT, immune-electron microscopy was employed according to Yang et al. [26]. Transgenic and wild-type cells were centrifuged at 3000×*g* for 10 min. The specimens were fixed with 2 % paraformaldehyde and 2 % glutaraldehyde in 0.1 M phosphate buffer (pH 7.2) under vacuum for 20 min and followed by incubation at room temperature for 3 h. The steps of thin sectioning and immuno-electron microscopy were performed as described previously. The sections were photographed by JEM-2000EXII electron microscope (JEOL, Japan) operating at 80 kV.

## Abbreviations

DAG: diacylglycerol
EM: electron microscopy
GC–MS: gas chromatography-mass spectroscopy
GPAT: glycerol-3-phosphate acyltransferase
LPAAT: lysophosphatidic acid acyltransferase
LysoPA: lysophosphatidic acid
MUFA: monounsaturated fatty acid
PA: phosphatidic acid
PUFA: polyunsaturated fatty acid
SFA: saturated fatty acid
TAG: triglycerol
